# 3D Culture in Functionalized FN‐Silk Networks Facilitate Proliferation, Differentiation and Phenotypic Stability of Mature Human Primary Cells and Stem Cells

**DOI:** 10.1002/bit.70002

**Published:** 2025-06-17

**Authors:** Astrid Källén, Nayere Taebnia, Mona Widhe, Volker M. Lauschke, My Hedhammar

**Affiliations:** ^1^ Division of Protein Technology School of Biotechnology, KTH Royal Institute of Technology Stockholm Sweden; ^2^ Spiber Technologies AB Stockholm Sweden; ^3^ Department of Physiology and Pharmacology Karolinska Institutet Stockholm Sweden; ^4^ Center for Molecular Medicine Karolinska Institutet and University Hospital Stockholm Sweden; ^5^ Dr. Margarete Fischer‐Bosch Institute of Clinical Pharmacology (IKP) Stuttgart Germany; ^6^ University of Tübingen Tübingen Germany; ^7^ Department of Pharmacy The Second Xiangya Hospital, Central South University Changsha China

**Keywords:** 3D culture, adipogenic differentiation, FN‐silk network, primary hepatocytes

## Abstract

The recombinant functionalized silk protein FN‐silk, including a cell adhesion motif from fibronectin, can form networks suitable for 3D culture of adherent cells. Such FN‐silk networks have previously been shown to support the growth and differentiation of a wide array of cell types. Herein, we have developed a user‐friendly methodology for the creation of free‐floating FN‐silk networks in 96‐well plates with both mature human primary cells and stem cells. We show that human mesenchymal stem cells (hMSC) cultured in FN‐silk networks form both cell‐cell and cell‐matrix contacts, resulting in tissue‐mimicking 3D cultures. Viability and expression analysis revealed that hMSC in FN‐silk networks have an initial proliferative phase with high cell viability and significantly lower hypoxia and apoptosis, compared to when cultured as scaffold‐free spheroids. The FN‐silk networks were shown to support differentiation of hMSC into adipocyte‐like cells with well‐maintained viability during the 3‐week‐long differentiation period, in contrast to the very poor long‐term viability of scaffold‐free 3D cultures. Improved adipogenesis was confirmed by lipid droplet staining, quantification of intracellular triglycerides, and secreted adiponectin levels, as well as expression analysis of multiple *bona fide* adipose markers. Lastly, we show that primary human hepatocytes maintain important functions and phenotypic markers when cultured in FN‐silk networks, features that are lost rapidly during conventional 2D culture. We therefore propose FN‐silk networks as a valuable scaffold for 3D human cell cultures, providing support for cell proliferation, differentiation, and the maintenance of critical tissue‐specific functionality.

## Graphical abstract

The authors present a new technique for 3D culture of mammalian cells with the support of a microfiber‐based network of fibronectin (FN)‐functionalized recombinant silk. This feasible 2‐step method results in highly viable free‐floating 3D cultures with tissue‐mimicking morphology. The method allows for differentiation of stem cells, as well as maintaining key functions of specialized human cells, that are easily lost in conventional 2D culture.

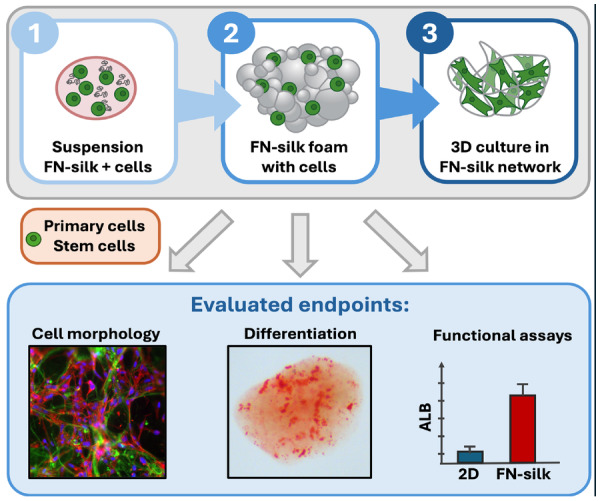

## 1 Introduction

Cell‐based In Vitro models are utilized extensively in biomedical research for translational applications (Youhanna et al. 2022). However, the models used often have limited physiological relevance. Some examples of important In Vitro models are tumor models for cancer research and anticancer drug testing (Barbosa et al. 2022), liver models for studying drug metabolism and hepatotoxicity (Lauschke et al. 2019; Yang et al. 2023), as well as adipose tissue models and their precursor cells for studying adipogenesis and related diseases such as obesity and diabetes (Armani et al. 2010; Ruiz‐Ojeda et al. 2016). For all such models, it is essential to use an appropriate cell source as well as culture method to mimic the In Vivo counterpart as closely as possible. Otherwise, the reduced physiological relevance and resulting translational gaps contribute majorly to the high failure rates in drug development today (Krüger and Kopp 2023; Langhans 2018).

Primary cells, isolated directly from the tissue of interest, are the gold standard cell source for obtaining physiologically relevant models. However, primary cells are often sensitive and difficult to culture, especially for longer periods, that is, multiple days or weeks. Consequently, although the starting material is of high physiological relevance, these cells can rapidly de‐differentiate, get reduced functionality and viability, which can hamper their utility for translational applications. Another important cell source is self‐renewing stem cells that can be differentiated into specialized cells of interest (Zakrzewski et al. 2019). Stem cells can be highly useful to study for example, development, differentiation pathways, and certain diseases, as well as to model various tissues or organs (Clevers 2016; Wu and Izpisua Belmonte 2016). Multipotent stem cells, such as mesenchymal stem cells (MSCs), can be differentiated into a limited number of cell types, including osteoblasts, chondrocytes, and adipocytes (Assis‐Ribas et al. 2018; Dominici et al. 2006).

In addition to the type of cell, the culturing method itself also plays a crucial role in shaping molecular phenotypes and functions. Conventionally, adherent cells are cultured as monolayers on a two‐dimensional (2D) surface. While many scientific discoveries have been made using this culture paradigm, 2D cultured cells rapidly deteriorate and differ substantially from the natural physiological environment where cells grow in a three‐dimensional (3D) context and have contacts with both other cells and the surrounding extracellular matrix (ECM) (Jensen and Teng 2020; Langhans 2018). More complex culturing methods in 3D are thus deemed necessary. A notable example highlighting the importance of the organotypic culture methods is primary human hepatocytes (PHH), the most common cell type in the liver, which plays critical roles in drug metabolism, for studying hepatotoxicity and modeling other types of liver diseases. PHHs have previously been reported to lose important hepatic functions and de‐differentiate after only hours in 2D culture (Lauschke et al. 2016), but can be retained for many weeks in 3D culture as self‐assembled spheroids with maintained hepatic functions (Bell et al. 2016).

Spheroid culture is a widely used method that entails the self‐assembly of cells arranged into sphere‐like structures, typically formed by promoting cell‐cell adhesion, for example, in ultra‐low attachment plates (Ingelman‐Sundberg and Lauschke 2022; Ryu et al. 2019). Cells in scaffold‐free spheroids have extensive cell‐cell contacts and, but limited, cell‐matrix contacts provided solely from the endogenous ECM components the cells produce themselves. However, formation of scaffold‐free spheroids often requires optimization of culture conditions to obtain uniform spheroids (Langhans 2018). Some cell types form loose structures, while other cell types, particularly highly proliferating cells, form more dense spheroids, which, with increasing sizes, will limit the diffusion of oxygen and nutrients to the center, thereby causing hypoxia and formation of necrotic cores (Langhans 2018; Pinto et al. 2020). Spheroids have therefore been claimed to represent tissues with natural hypoxic cores, such as tumor models (Chen et al. 2023; Langhans 2018; Petrova et al. 2018; Pinto et al. 2020). Most normal healthy tissues, however, contain both a supporting ECM that allows a less compact arrangement and have tightly regulated vasculature with capillary networks providing sufficient oxygen and nutrients to keep the tissue viable and functional (Asthana and Kisaalita 2012).

To circumvent the aforementioned issues associated with spheroid culture of proliferating cells, it would be desirable to have an ECM‐mimicking matrix that allows cell‐matrix contacts and sufficient diffusion of oxygen and nutrients to the core while still maintaining the beneficial 3D context and cell‐cell contacts. The approach that we explore herein is to incorporate the 3D cultured cells in an ECM‐like network made of a recombinantly produced silk protein engineered to contain the RGD‐containing cell binding motif from fibronectin (FN) (Widhe et al. 2016). The resulting FN‐silk protein is 23 kDa, with the silk part derived from the sequence of the dragline silk protein major ampule spidroin 1 (Stark et al. 2007). FN‐silk in solution self‐assembles at interfaces under physiological conditions (Nilebäck et al. 2018), which can be utilized to form various FN‐silk formats such as fibers (Kvick et al. 2021), membranes (Gustafsson et al. 2020; Hjelm et al. 2023; Tasiopoulos et al. 2021), and networks (Åstrand et al. 2020; Collodet et al. 2023; Johansson et al. 2019) suitable as matrices for cell culture in different applications.

We have previously reported that FN‐silk networks can be successfully used to culture a wide array of cell types including for example, pluripotent stem cells (Åstrand et al. 2020; Fiorenzano et al. 2021), MSC (Johansson et al. 2019), breast cancer cell lines, and primary breast cancer tumor cells (Collodet et al. 2023). Furthermore, cells have been demonstrated to proliferate and attach to FN‐silk networks with elongated cell morphologies, defined focal adhesion points, and high cell viability for long culture periods (Johansson et al. 2019). Moreover, successful differentiation of pluripotent stem cells to neuronal lineages (Åstrand et al. 2020) and brain organoids (Fiorenzano et al. 2021; Sozzi et al. 2022) supported by FN‐silk networks has been described, as well as differentiation of MSCs to adipogenic and osteogenic lineages (Johansson et al. 2019; Widhe et al. 2022). Rheology analysis of larger FN‐silk networks has shown that the scaffolds exhibit both a high shear stress elasticity and a striking capacity to maintain structural integrity under dynamic load conditions (Gkouma et al. 2024). Even after longer In Vitro cultures, up to 4 months, no signs of degradation of the FN‐silk structures have been seen (Johansson et al. 2019; Sozzi et al. 2022), likely due to the β‐sheet rich structures of silk protein sequence lacking typical sites for proteolytic degradation (Hedhammar et al. 2008).

Here, we present an updated, user‐friendly protocol for producing free‐floating FN‐silk networks in 96‐well format for simplified handling of 3D cultures of adherent human cells in several replicates. We have herein characterized the cells growing in the networks with regard to morphology, cell viability, and differentiation. The free‐floating FN‐silk networks provided good viability of stem cells during expansion as well as the longer culture period needed for differentiation. In addition, we demonstrate that this new FN‐silk format can improve the preservation of specific phenotypes and functions of primary human cells in long‐term cultures, using PHH as an example.

## 2 Results and Discussion

### 2.1 FN‐Silk Networks With Cells Form Free‐Floating 3D Cultures

**Figure 1:**
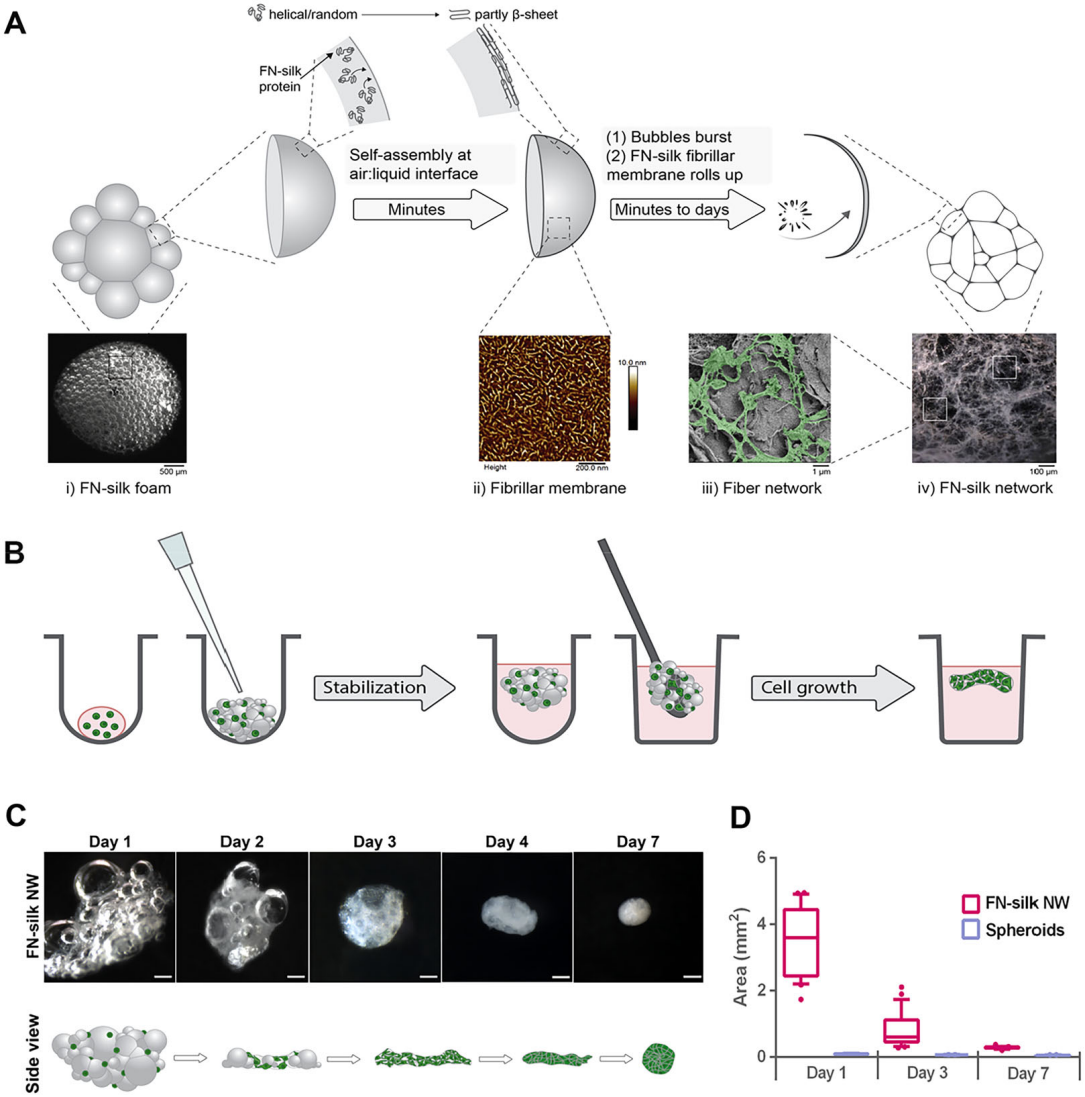
Generation of the FN‐silk network 3D culture format. (A) Illustration of the transformation from FN‐silk foam (i) to network (iv). The soluble FN‐silk protein self‐assembles at the air liquid interface of each air bubble, in a process during which the protein structure undergoes transformation from helical/random to partly β‐sheet structures (Nilebäck et al. 2018). The initial air bubbles are enclosed by fibrillar silk membranes (ii, AFM image reproduced from [Nilebäck et al. 2018]) that eventually break and create thin FN‐silk fibers (iii, green‐colored structures) building up the 3D FN‐silk network (iv). (Cells are not visualized here.) (B) Schematics of the protocol for creation of FN‐silk networks. A mix of cell suspension (cells in green) and soluble FN‐silk is added as a single droplet per well in a U‐bottom ULA‐plate. Small air bubbles are introduced in the droplet by pipetting, to create dense FN‐silk foams with integrated cells. The foam 3D structure is stabilized in a cell incubator followed by medium addition to detach the foams from the wells. The free‐floating foams are thereafter transferred to new wells in a flat bottom plate, before further culture in a cell incubator. Over time, as the cells grow and the membranes naturally burst, the foam is transformed to a free‐floating FN‐silk network with integrated cells. (C) Representative top‐view stereo light microscope images over 1 week of culture of free‐floating FN‐silk network 3D cultures, initially seeded with 10,000 hMSCs (*N* = 2, *n* = 10–24). Scale bars 300 µm. Side‐view illustrations of the 3D appearance over time of FN‐silk networks with integrated cells in green (lower panel). (D) Box plot graph (10%–90%) showing the size distribution (top‐view area measured by ImageJ Fiji software) of FN‐silk network 3D cultures (pink, *n* = 24) and scaffold free spheroids (violet, *n* = 24), both initially seeded with 10,000 hMSCs, during the first week of culture.

FN‐silk networks are created by introducing air bubbles into a FN‐silk solution. This results in the formation of a foam with very thin FN‐silk membranes around each air bubble (Figure 1a). Over time, these FN‐silk membranes burst and curl up to thin fibers creating a 3D network. The resulting FN‐silk networks mimic the ECM interstitial fibers due to similar dimensions (Figure 1a), mechanics (Gkouma et al. 2024), and decoration with the fibronectin‐derived cell adhesion motif (Johansson et al. 2019). Adherent cells can be directly integrated into the networks by simply adding a cell suspension to the soluble FN‐silk before foaming. This concurrent cell integration during FN‐silk assembly provides a homogeneous cell distribution throughout the scaffold (Collodet et al. 2023; Johansson et al. 2019). Additionally, FN‐silk can easily be combined with other natural ECM components, such as laminins, by including them in the FN‐silk/cell suspension during foaming (Åstrand et al. 2020). Herein we have used this principle to develop a novel protocol for 3D cell culture in free‐floating FN‐silk networks in 96‐well plates (Figure 1b).

FN‐silk‐supported 3D cultures were created by adding a droplet containing a mixture of soluble FN‐silk protein and cells to each well in a U‐bottom ULA‐plate, followed by rapid pipetting of air for about 10 s to introduce air bubbles and thereby initiate FN‐silk assembly. The FN‐silk protein has previously been shown to assemble into fibrils forming a membrane at the air‐liquid interface (Nilebäck et al. 2018). The process of self‐assembly can be expedited by subjecting the protein solution to shear forces (Kvick et al. 2021), in this case by the rapid pipetting. The duration of the pipetting has been optimized to yield a dense cluster of bubbles, each surrounded by a thin membrane of assembled FN‐silk fibrils. Prolonged pipetting leads to disintegration of the already build‐up structures. In contrast to previously published protocols (Åstrand et al. 2020; Collodet et al. 2023; Johansson et al. 2019), foaming was here conducted using a multichannel pipette in ULA‐treated 96‐well plates, which greatly facilitates handling of more replicates. Thereafter, the FN‐silk foams were briefly stabilized in either the LAF‐bench or cell incubator, followed by the addition of culture medium, which results in instantly detached free‐floating foams. For easier handling during culture, and to separate the foams from any non‐incorporated cells, the free‐floating foams were transferred to flat bottom plates using a lab spoon.

To characterize the macroscopic and microscopic morphology, FN‐silk networks with human mesenchymal stem cells (hMSCs) were prepared and cultured for 7 days. As evident from stereo microscope images (Figure 1c, corresponding for mMSCs in Supporting Information S1: Figure S1b), the FN‐silk foams with hMSC were gradually transformed over time. At Day 1, the FN‐silk appeared as a floating foam, still with several bubbles surrounded by a thin FN‐silk membrane (Figure 1c). As more and more of the FN‐silk membranes burst, thin fibers were observed, created from the ruptured and rolled‐up membranes, and together generating a fibrillar network (Day 2–3, illustrated in Figure 1a). With time, as the cells continued to grow, the 3D cultures with FN‐silk networks became more spherically shaped (Figure 1c, Day 4–7). The time needed for this transformation varies with different culture conditions such as with cell type, cell seeding number, and cell passage number (i.e., cell division rate). In contrast, when hMSCs were cultured as scaffold‐free spheroids (Supporting Information S1: Figure S1), dense structures were formed from start (day 1) and became smaller and seemingly more compact over time (Day 3 and Day 7) (Supporting Information S1: Figure S2). At Day 7, FN‐silk‐supported 3D cultures were larger and appeared to have a less compact internal structure, compared to scaffold‐free spheroids seeded with the same cell numbers (Figure 1d). We hypothesized that this less dense structure likely enables better diffusion of oxygen and nutrients to the inner core, and provides a better representation of the natural tissue morphology, compared to the more compact scaffold‐free spheroids. The final dimensions of the FN‐silk networks seem to be determined by several factors. First of all, the number of seeded cells and the amount of FN‐silk used, but also the size and shape of the vial (in this case 96 plate U shaped well) in which the foams are created and stabilized, affects the outcome. The nature of the cell type, for example, growth rate, morphology and propensity for adhesion to the FN‐silk fibers also seems to be decisive for the resulting dimensions. For some cell types, for example, mMSCs, we have observed a shape‐morphing behavior of the FN‐silk network cultures (Supporting Information S1: Figure S3b). This could possibly be of use in applications where the material needs to adapt to a certain cavity or in more complex biological systems (Mirzababaei et al. 2024). Taken together, the resulting 3D cultures in FN‐silk networks do vary somewhat in details, although the overall size and shape can be predicted. We regard this as closer to biological conditions, compared to any exact dimensions of pre‐fabricated scaffolds.

### 2.2 Protocol Options for Cell Addition and Air Bubble Removal

Our main strategy has been to have the cells present in the FN‐silk mix during foaming, to get immediately well‐distributed cells in the final culture. However, the cells can also be added to the FN‐silk directly after foaming if desired, for example, for very sensitive cells. This can be done by removing the medium after the initial foam detachment, and seeding the cells on top of the foams in a small medium volume (e.g., 5 µL), followed by an incubation to allow the cells to adhere properly before adding new medium. For the MSCs analyzed here, the foaming procedure does not seem to be harmful from a viability perspective, considering the good cell viability for hMSCs added before foaming, described in Section 2.4. Additionally, Live/Dead stainings comparing mMSCs seeded before and after foaming (Supporting Information S1: Figure S3b) show no clear difference in cell death. Possibly, addition of the cells after the foaming could lead to a more heterogeneous cell distribution within the 3D culture, or a less efficient cell capture into the network, the latter of which can be counteracted by increasing the seeding number.

FN‐silk foams naturally transform to networks when the membranes rupture and release the air bubbles, a process which usually takes 2–3 days to be completed. Especially for short term cultures, where the initial structure is of importance, it might be desirable that the bubbles disappear as soon as possible. We have thus investigated different ways to release the air bubbles, and thereby expedite the formation of the final network structure. One strategy is to perform a washing step (with e.g., PBS) followed by medium change during the first culture day(s), which promotes removal, or at least shrinkage, of air bubbles in the network (Supporting Information S1: Figure S4a). This is a straightforward method that can easily be implemented. We have also seen an indication that a mild shaking (95 rpm) during the incubation results in some air bubble removal (Supporting Information S1: Figure S4b). Moreover, as previously reported by Collodet et al. for FN‐silk foams with breast cancer cell lines made in 24‐well plates, air bubbles can be removed by applying a pressure difference to the foams using a 3D printed cap and a Vacusafe aspiration system (Collodet et al. 2023). Repeating this approach on foams with mMSCs showed some effect on air bubble removal, and also resulted in more homogeneous, but flatter, networks (Supporting Information S1: Figure S4c). The techniques for air bubble removal described here are optional, and were not included in the following herein presented experiments.

### 2.3 Cells Growing in FN‐Silk Networks Show Elongated Morphology Along the FN‐Silk Fibers

**Figure 2:**
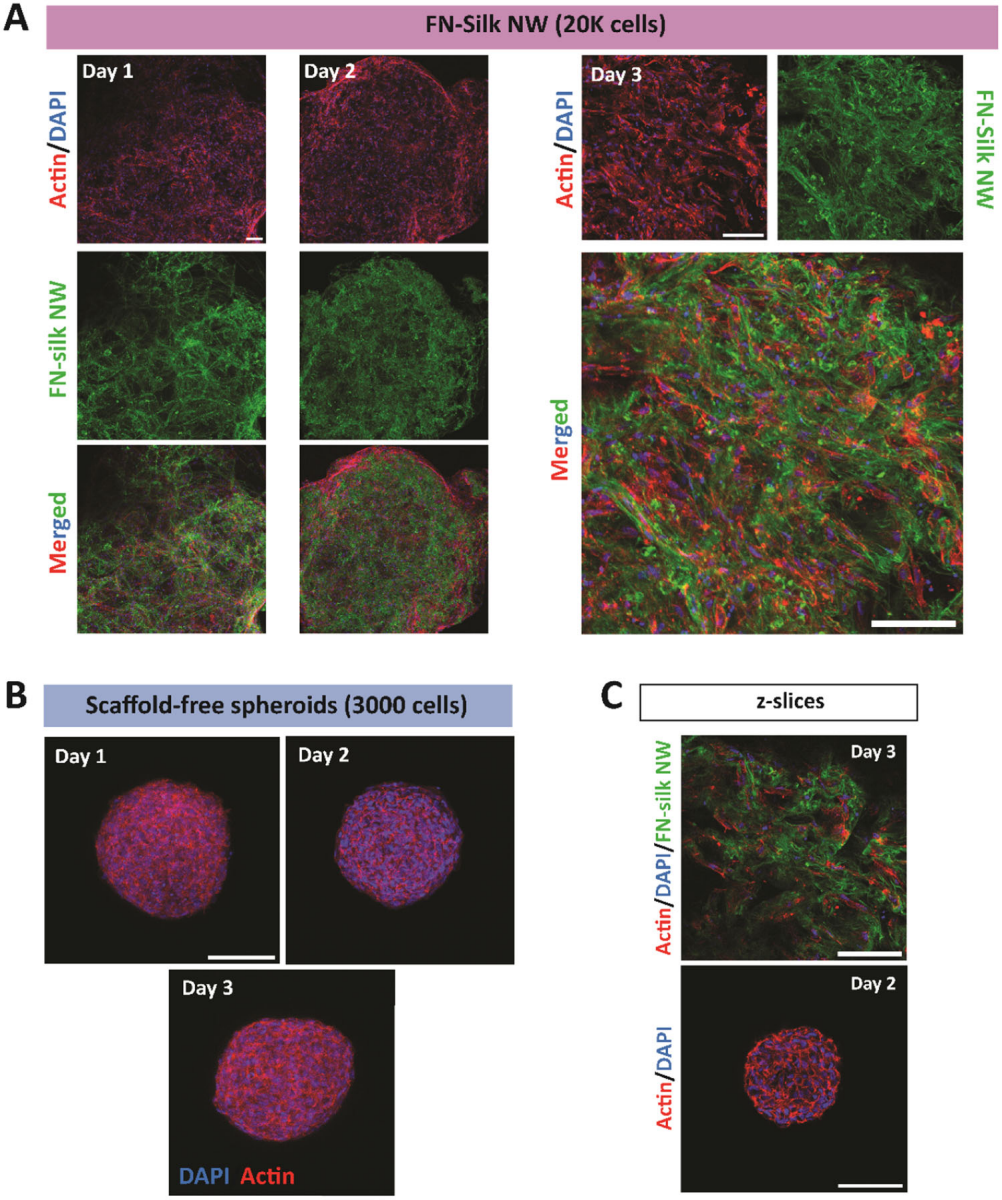
Microscopic view of cell morphology and matrix interaction in the 3D cell cultures. F‐actin (Alexa Fluor 647‐ Phalloidin, red) and nuclei (DAPI, blue) staining of (A) FN‐silk‐DyLight488 networks (green) initially seeded with 20,000 hMSCs, and (B) spheroids seeded with 3000 hMSCs, as imaged by confocal microscopy (*N* = 2, *n* = 2). (C) Representative z‐slices of FN‐silk network and spheroid with hMSCs. Scale bars: 100 µm.

To further analyze the internal morphology, hMSCs were cultured in FN‐silk networks and as spheroids for various time points before fixation and staining for F‐actin and nuclei. To visualize the FN‐silk, a small fraction of fluorescently labeled FN‐silk was included in the networks. Imaging with confocal microscopy showed that the FN‐silk networks seemingly were composed mainly of thin fibers and sheets, and that the cells grew with elongated morphology along the FN‐silk, but still connected to each other (Figure 2a). This suggests the presence of both cell‐matrix and cell‐cell contacts within the FN‐silk network culture, similar to cells growing in the natural ECM environment. Moreover, there was still some room left for further cell expansion during the first 2 days of culture. Around Day 3, cultures in FN‐silk showed high cell confluency, with fewer empty spaces left (Figure 2a, right panel). In contrast, the spheroids had formed cell‐dense structures already at Day 1, seemingly with more rounded cells compared to in the networks, as no elongated cells could be spotted in the inner parts of the culture (Figure 2b). This is in agreement with previous reports showing that scaffold‐free hMSC spheroids have mainly round and irregular cells in the interior, and some flattened and elongated cells at the surface (Bartosh et al. 2010; Sart et al. 2014).

An important practical advantage of FN‐silk networks compared to conventional scaffold‐free spheroids is the simplified handling during culture. Complete medium changes can relatively easily be carried out in standard flat‐bottom culture plates without deforming the cultures. This is usually very difficult for scaffold‐free spheroids due to their small size and fragility, and requires the use of for example, half‐volume medium changes, expensive special plates or other methods to facilitate handling. Moreover, the formation of 3D cultures with the FN‐silk network approach is not as cell‐type dependent as with scaffold‐free methods, for which some cell types form compact spheroids while other cell types may form very loose aggregates that barely hold together. Both mouse and human MSCs, of varying passage numbers (22–36 for mMSC, 3–9 for hMSCs) and seeding densities (10,000–20,000) have been successfully integrated into FN‐silk networks and formed coherent 3D cultures, although with slight variations in how dense the cultures became.

### 2.4 FN‐Silk Networks Support Proliferation and High Viability

**Figure 3:**
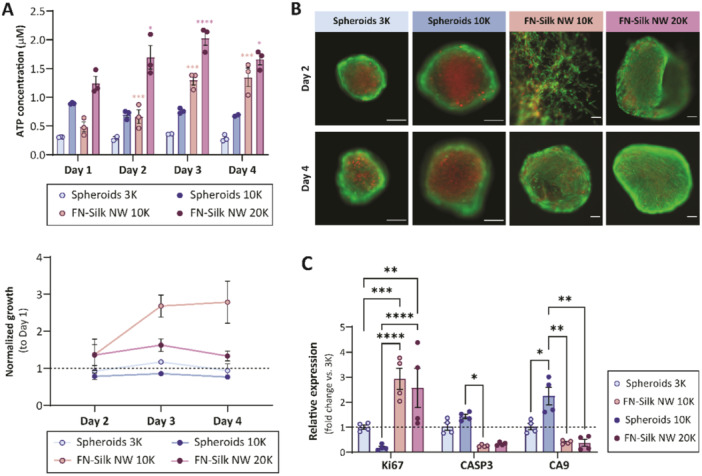
Viability of hMSCs in FN‐silk networks and spheroids. (A) ATP levels (*n* = 3) as measured by CellTiter‐Glo 3D Cell Viability assay of hMSCs in FN‐silk networks initially seeded with 20,000 (purple), or 10,000 (beige) cells, and spheroids initially seeded with 10,000 (dark blue) or 3000 (light blue) hMSCs. Indicated p‐values show significant differences in comparison to day 1 for respective condition. Data is representative of three independent experiments. (B) Live (calcein, green) and dead (ethidium homodimer‐1, red) staining of FN‐silk networks seeded with 20,000 (*n* = 3) or 10,000 (*n* = 3) hMSCs, and spheroids seeded with 10,000 (*n* = 3) or 3000 (*n* = 3) hMSCs after 2 and 4 days of culture, as imaged by fluorescence microscopy. Images are representative of two independent experiments. Scale bars 100 µm. (C) Relative expression of mRNA markers for proliferation (Ki67), apoptosis (CASP3) and hypoxia (CA9) in FN‐silk networks initially seeded with 20,000 (purple bar, Day 2), or 10,000 hMSCs (beige bar, Day 3) and spheroids started with 10,000 (dark blue, Day 2) or 3000 hMSCs (light blue bar, Day 3), as measured by qPCR (*n* = 2). Values presented as relative to spheroids with 3000 hMSCs. *p* values indicated with *p < 0.05, **p < 0.01, ***p < 0.001 and ****p < 0.0001.

The viability of hMSCs cultured in FN‐silk networks was assessed and compared to scaffold‐free spheroids using the metabolic activity assay CellTiter‐Glo, specifically developed for 3D cultures, and live/dead fluorescent staining. CellTiter‐Glo 3D analysis (Figure 3a) showed that the metabolic activity of hMSCs cultured in FN‐silk networks increased from Day 1 until Day 3, followed by a plateau or slight decrease for networks seeded with 10,000 or 20,000 cells, respectively. This indicates a proliferative phase during the first days, which slows down when high cell densities are reached within the FN‐silk networks around Day 3. This is in line with the described micrographs (Figure 2), where available space for cell growth was observed on Days 1 and 2, while higher confluency was seen on Day 3. In contrast, the scaffold‐free spheroids had relatively constant metabolic activity during the first 4 days of culture, both for 3000 and 10,000 cells/well, indicating low or non‐existent proliferative activity and decreasing cell viability in the lack of FN‐silk. Similar metabolic changes during 3D culture were observed with mouse mesenchymal stem cells (mMSC) (Supporting Information S1: Figure 3a). Here, a fourfold increase in ATP concentration was observed from Day 1 to Day 4 for mMSCs growing in FN‐silk networks, whilst spheroids instead showed a marked decrease in metabolic activity, even when only 3000 cells per well were used.

Notably, the metabolic activity of hMSCs after seeding 10,000 cells per FN‐silk network was significantly lower (*p < 0.05) at day 1 compared to spheroids from the same number of cells. This observation suggests that some cells might be lost during the foaming step. In contrast, for mMSCs, the metabolic activity in FN‐silk networks was higher (*p < 0.05) at Day 1 compared to spheroids seeded with the same cell numbers. This discrepancy between the different cell types is likely due to the higher proliferation rate of mMSCs in networks or other cell type‐specific properties, such as proneness to adhere, or different medium components that might affect the cell capture efficiency in the FN‐silk networks.

As a complement to the metabolic activity measurements, Live/dead staining was performed at multiple time points after seeding. Also here, superior cell viability was seen in FN‐silk networks compared to spheroids (Figure 3b). Whereas scaffold‐free spheroids exhibited cores with dead cells already at Day 2, especially at higher seeding numbers, FN‐silk supported cultures exhibited high viability with only a few dead cells present at both Day 2 and Day 4, independent of initial seeding numbers. The three‐dimensionality of the cultures makes imaging challenging, since it is not possible to focus on all cells at the same time. However, after careful microscopy analysis with different focus, the absence of a necrotic core in the FN‐silk networks was evident, regardless of culture day as well as culture density, suggesting that even in denser FN‐silk network cultures, the supply of oxygen and nutrients should be sufficient also to the inner cells, supposedly provided by the presence of FN‐silk fibers throughout the 3D culture.

To investigate the difference in viability of cells in FN‐silk networks and spheroids closer, qPCR was conducted with markers for apoptosis, hypoxia and proliferation at culture Day 2 and 3 (Figure 3c). The results showed a significantly higher expression for the proliferation marker Ki67 for cells in FN‐silk networks compared to scaffold‐free spheroids. Moreover, the expression of the apoptosis marker CASP3 and the hypoxia marker CA9 were significantly higher (*p* ≤ 0.05 and 0.01 respectively) in scaffold‐free spheroids initially seeded with 10,000 cells compared to the FN‐silk cultures. These results thereby further support our hypothesis that the less compact 3D structure provided by the FN‐silk, possibly together with the free‐floating nature of the FN‐silk network, helps to improve the oxygen supply, increase access to nutrients in the culture medium, and facilitate exchange of carbon dioxide and cellular waste. Consequently, the viability throughout the 3D culture is improved within the FN‐silk network. The reason for the increased cell viability seen in the FN‐silk networks compared to the scaffold‐free spheroids is likely the previously demonstrated improved accessibility of oxygen and nutrients to the inner core of the culture (Sozzi et al. 2022).

The beneficial properties discovered so far encouraged us to go on to investigate two possible applications of the free‐floating FN‐silk networks, namely stem cell differentiation and hepatocyte culture.

### 2.5 FN‐Silk Networks Support Adipogenic Differentiation of hMSCs

**Figure 4:**
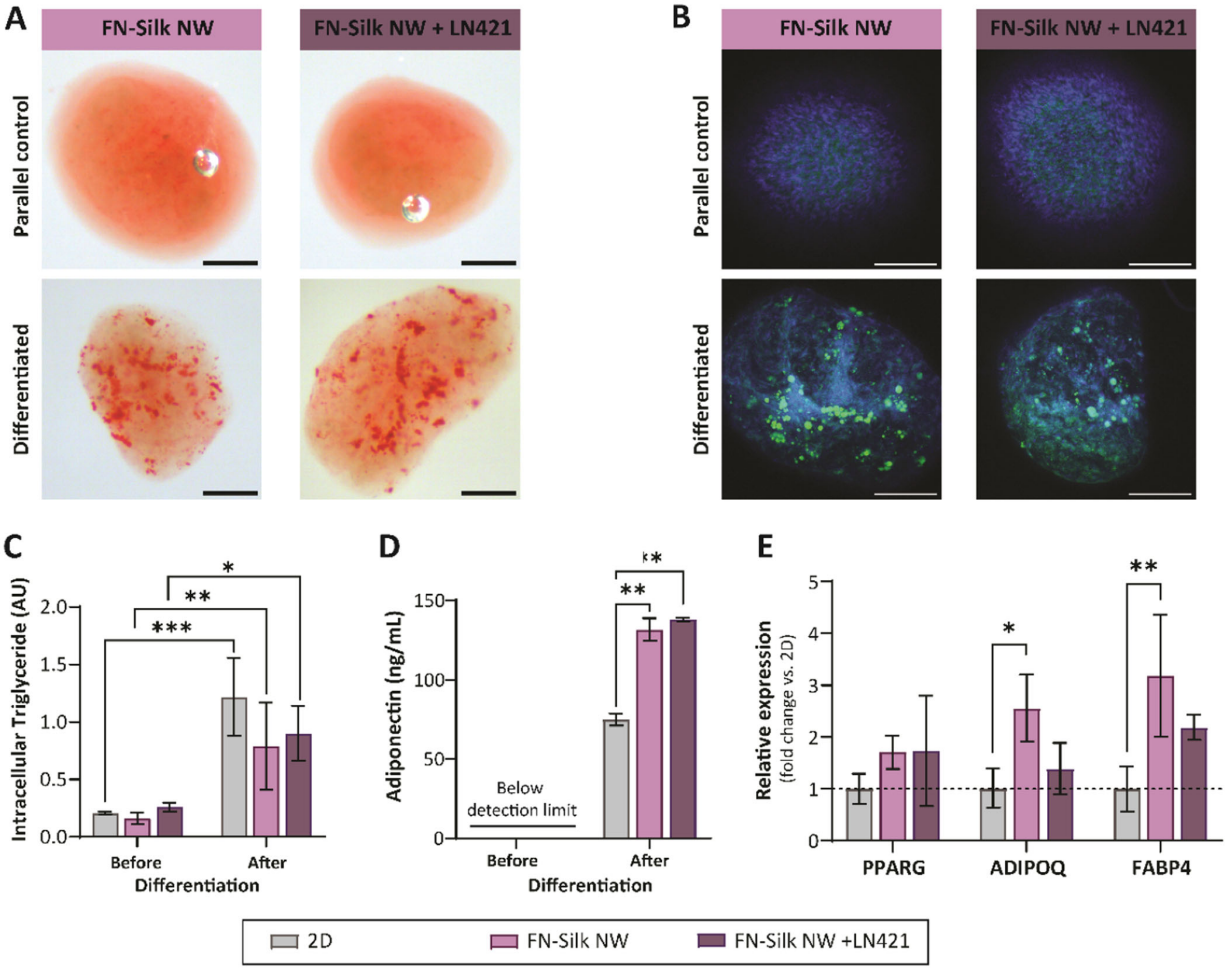
Adipogenic differentiation of hMSCs in FN‐silk networks. FN‐silk network cultures without (left) or with (right) laminin 421, stained for intracellular lipid droplets with (A) Oil Red O (Red) (*n* = 3) and (B) BODIPY ((4,4‐difluoro‐3a,4adiaza‐s‐indacene, green) with DAPI nuclear counterstain (blue) (*n* = 3) after 21 days of adipogenic differentiation (differentiated, bottom panel). Parallel controls that were cultured without the addition of adipogenic factors for 21 days, are shown for comparison (upper panel). Scale bars 200 µm. (C) Lipid quantification with AdipoRed detecting triglycerides in dissociated cells from FN‐silk networks and monolayers before and after adipogenic differentiation. Detected levels normalized to cell numbers of respective culture are shown as mean +/‐ std (*n* = 3). (D) Adiponectin secretion in supernatants before and after differentiation of hMSC in FN‐silk network 3D cultures with and w/o Laminin 421 and 2D controls (*n* = 3), (E) Relative mRNA expression of the adipogenic markers PPARG, ADIPOQ and FABP4 in FN‐silk network 3D cultures after adipogenic differentiation as measured by qPCR. Levels were normalized to 2D cultures. (*n* = 3). P‐values indicated with *p < 0.05, ** *p* < 0.01 and ***p < 0.001. ADIPOQ, adiponectin; FABP4, fatty acid binding protein 4; NW, network; N.D., not detectable; LN, laminin; PPARG, peroxisome proliferator‐activated receptor gamma.

To evaluate if the presented FN‐silk network culture format can support differentiation of human stem cells, we decided to test whether multipotent hMSCs cultured in FN‐silk networks can be differentiated to adipocytes by culturing the networks in adipogenic differentiation medium. Since laminins with the α4 chain, such as LN411 and LN421, have been shown to be upregulated during adipogenic differentiation and are present in the ECM surrounding mature adipocytes (Noro et al. 2013; Vaicik et al. 2014), we decided to include LN421 in the FN‐silk networks, as an additional condition, to investigate if its presence would have a positive effect on the differentiation process. The addition of laminins has previously been shown to not affect the ability of FN‐silk to form a network with integrated cells (Åstrand et al. 2020). We first determined the culture time required for the cells to reach a proper density within the FN‐silk network cultures. For 2D cultures, a confluency of 80%–90%, recommended by the differentiation medium manufacturer (PromoCell) for induction of adipogenesis, was used. For 3D cultures, it is more challenging to determine the confluence. Therefore, we instead used the CellTiter‐Glo 3D results, together with the F‐actin and DAPI stainings of hMSCs growing in the networks. Evident from these data, cells were populated throughout the FN‐silk network at Day 3 (Figure 2), exhibiting proper cell‐cell contacts. Moreover, the cells reached a peak in metabolic activity around Day 3 (Figure 3a), indicating a high confluency. Based on this, the differentiation was started on Day 3 (Figure 4 and Supporting Information S1: Figure S5) or Day 4 (Supporting Information S1: Figure S6a) for FN‐silk cultures seeded with 20,000 cells. Properties such as proliferation rates can, however, vary between cell batches and passages, which entails that the starting point for the induction of differentiation might require adjustment.

After the 3 weeks long differentiation period, four types of analysis were used to assess if the hMSCs had been successfully differentiated to adipocytes in the FN‐silk networks and 2D controls; lipid droplet stainings, triglyceride quantification, quantification of secreted adiponectin with ELISA, and expression analysis of adipogenic markers. Moreover, the viability of 3D cultures was assessed using CellTiter‐Glo 3D and Live/Dead staining (Supporting Information S1: Figure S5). CellTiter‐Glo analysis showed that less than 5% of the metabolic activity of the spheroids (3000 hMSCs) remained after 20 days of differentiation compared to the differentiation start, while corresponding number for networks were above 25%. Live/Dead stainings confirmed that the cells in the spheroids were mainly dead, while cells in the FN‐silk networks still had relatively good viability also at the end of the differentiation period. Due to extremely low viability over the culture time needed, and difficulties in handling during culture and analysis, scaffold‐free spheroids were excluded from further differentiation experiments.

Adipocytes can easily be distinguished morphologically by the presence of intracellular lipid droplets for storage of triglycerides. Lipid droplet staining using the lipid stains Oil Red O (Figure 4a) and Bodipy (Figure 4b) were thus performed on differentiated samples and on parallel controls cultured in growth medium. Lipid droplets were observed in FN‐silk supported cells after differentiation, but not in the parallel control. The droplets were detected throughout the 3D structure in varying sizes and numbers. Similar appearance was observed for FN‐silk networks with additional LN421 (Figure 4a,b, Supporting Information S1: Figure S6a). The cells differentiated on 2D also had some lipid droplets, although of seemingly smaller size, and only present in a few areas of the monolayers (Supporting Information S1: Figure S6b,c).

To further quantify the presence of intracellular triglycerides, the AdipoRed assay was used to analyze triglyceride levels in cell suspensions from trypsinized cultures in FN‐silk networks and 2D (Figure 4c). The results showed a significant increase in triglyceride levels after differentiation of cells cultured both in FN‐silk networks and as monolayers, compared to before differentiation. No significant difference was observed between the different culture formats, or by the addition of LN421. However, the comparison between culture formats should be considered semi‐quantitative since adjustments of the protocol was required to release the cells from the FN‐silk networks, which may add uncertainties to the measured values. The results still indicate increased triglyceride levels following adipogenic differentiation in the investigated culture formats, supporting the presence of adipocytes described above.

Another important feature of mature adipocytes is their ability to secrete the hormone Adiponectin, which can be measured from the culture supernatant. Samples collected before differentiation were below the level of detection, whereas a marked increase could be detected for all culture conditions after adipogenic differentiation (Figure 4d). Since adiponectin is produced specifically by adipose tissue (Martella et al. 2014), the presence of secreted adiponectin indicates that adipogenic differentiation has occurred for all formats. Although the total number of cells present in each culture format was not controlled for, the significantly higher concentrations of adiponectin detected in supernatants from differentiated FN‐silk cultures, as compared to monolayers (*p* ≤ 0.01), is indicative of a successful differentiation in the FN‐silk networks, with or without LN421.

Finally, mRNA expression analysis using qPCR, detected the adipogenic markers PPARG, ADIPOQ and FABP4 in cells differentiated in FN‐silk networks or as monolayers (Figure 4e). The expression of ADIPOQ and FABP4 was significantly higher for cells in FN‐silk networks without LN421 compared to 2D control (*p* ≤ 0.05 and *p* ≤ 0.01, respectively). The onset of these genes is a clear sign of adipogenic differentiation, and the results also suggest that the differentiation process is more efficient in FN‐silk network cultures, as compared to standard monolayers.

Altogether, our findings show that the developed FN‐silk network protocol can support adipogenic differentiation of hMSCs. Moreover, the differentiation is seemingly more efficient compared to conventional 2D cultures and gives considerably higher viability compared to scaffold‐free spheroids.

### 2.6 FN‐Silk Networks Support the Long‐Term Maintenance of Large Functional 3D Cultures of Primary Human Hepatocytes

**Figure 5:**
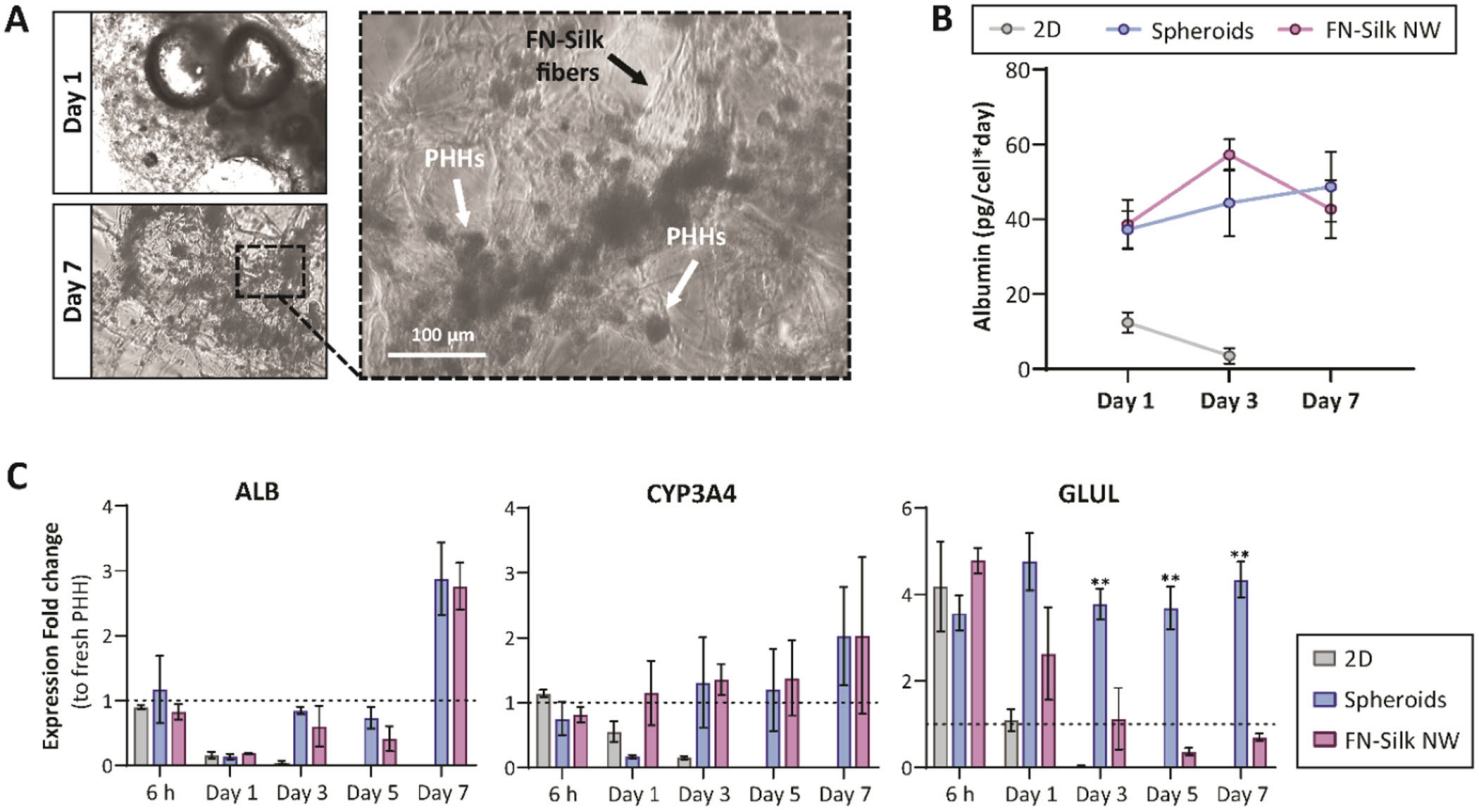
Culture in FN‐silk networks maintain the functionality of primary human hepatocytes. (A) Phase contrast micrographs of primary human hepatocytes (PHHs) on Day 1 (top) and Day 7 (bottom) of culture in FN‐silk. The inset shows the interaction between the PHHs and FN‐silk fibers. (B) Albumin secretion, normalized to cell number, over one week in different culture formats (*n* = 3 per time point). (C) mRNA expression levels of key functional markers in PHHs: ALB, CYP3A4, and GLUL. The expression of ALB and the drug‐metabolizing enzyme CYP3A4 in the FN‐silk culture format remains similar to 3D spheroids (the standard In Vitro model), whereas in 2D culture, the expression of these markers significantly declines after 24 h. GLUL expression exhibits a distinct pattern in FN‐silk compared to scaffold‐free spheroids, likely due to differences in oxygen levels between the two culture formats (*n* = 3).

Next, we assessed the culture of primary human hepatocytes (PHHs) in FN‐silk networks, as a representative of a highly sensitive primary human cell type. With this approach, we also wanted to investigate if 3D culture in FN‐silk could help preserving hepatocyte key features that are rapidly lost during standard 2D culture of PHHs (Lauschke et al. 2016). We evaluated PHHs both morphologically and functionally over 7 days in culture and compared their performance to standard 2D monolayers and well‐characterized 3D scaffold‐free spheroid cultures (Bell et al. 2017; 2018; Vorrink et al. 2017). The time point 7 days was selected since previous investigations using PHHs have shown that 7 days in culture is required for the aggregation into spheroids (Bell et al. 2017, 2018; Vorrink et al. 2017), even though FN‐silk networks are formed immediately and generally reach stable state without remaining bubbles already a few days after seeding (see section 2.3 and 2.4). The culture of PHHs in FN‐silk ( ~ 15,000 cells/network) resulted in loose and interconnected networks of consistent size, enabling efficient nutrient and oxygen diffusion to all cells. After 1 week in culture, the formed cell clusters exhibited a homogeneous morphology with well‐defined cell boundaries and no evident necrosis (Figure 5a).

During culture, the cells in both FN‐silk networks and scaffold‐free spheroids underwent compaction due to increased cell‐cell contacts, which correlates with increased functionality as previously reported (Oliva‐Vilarnau et al. 2020). Hepatocyte‐specific functionality was assessed by measuring albumin secretion, which remained stable for cells in both FN‐silk networks and scaffold‐free spheroids throughout the culture period of 7 days (Figure 5b). In contrast, hepatocytes cultured in a 2D monolayer rapidly de‐differentiated, as evidenced by a sharp decline in albumin secretion already after 24 h of culture.

We further evaluated mRNA expression levels of key functional markers, including a secreted hepatic factor (*ALB*), the drug‐metabolizing enzyme (CYP3A4), and the oxygen‐sensitive gene glutamine synthetase (GLUL) (Halpern et al. 2017; Tonon et al. 2019). PHHs in FN‐silk networks exhibited a similar expression pattern to scaffold‐free spheroids for ALB and CYP3A4 over the 7‐day culture (Figure 5c). However, GLUL expression was significantly lower for PHHs in FN‐silk networks from Day 3, when the network is stabilized and the air bubbles from the foam are gone. Importantly, GLUL expression is highly expressed only in pericentral hepatocytes (Ben‐Moshe and Itzkovitz 2019), suggesting that cells in scaffold‐free spheroids experience lower partial oxygen pressure (pO₂) compared to those in FN‐silk networks, which retain sufficient nutrient supply despite the considerably larger sizes. This is striking, since liver spheroids comprising more than 10,000 cells typically exhibit rapid declines in cell health and the formation of necrotic cores, likely due to the limited oxygen and nutrient supply. Consequently, these results indicate that FN‐silk networks support the formation of larger 3D structures in which PHH remain well‐oxygenated over 7 days, likely due to the accessible, loose, and floating structure. This system preserves native cellular phenotypes to a higher degree compared to conventional monolayer culture, and the cultured primary cells retain their physiological functionality similar to In Vivo conditions. Moreover, by providing the opportunity to skew hepatocellular phenotypes towards pericentral identities, FN‐silk networks might be useful for assessing the distribution of hepatocyte functions across the different zones (Wesseler et al. 2023).

## 3 Conclusion

We herein demonstrate high cell viability, proliferation, and differentiation of various cell types within free‐floating 3D cultures supported by FN‐silk networks. Specifically, these networks were shown to support the differentiation of human stem cells in 3D and proved suitable for the long‐term culture of primary human hepatocytes with mature molecular phenotypes and functions.

Compared to scaffold‐free spheroids, the procedure to make the FN‐silk networks with integrated cells requires some extra steps (pipetting, incubation, addition of media, and transfer to a new well), which takes a bit of extra time during the preparation phase and might demand some practical training for reproducible results. However, once formed, the FN‐silk network cultures are stable and easy to handle during media change etc. Further analysis, such as staining procedures, embedding and so forth, is thereby much facilitated compared to when working with scaffold‐free spheroids. Herein, we used 96‐well plates for the 3D cultures, which were prepared using an 8‐channel multipipette. The protocol has also been adapted to 384‐well plates, but smaller 3D cultures than that are difficult to prepare with FN‐silk networks, since this procedure requires the formation of air bubbles. For the FN‐silk network cultures to reach a suitable confluence, a certain ratio of cells needs to be seeded, even for the smaller culture volumes. However, cells in the free‐floating FN‐silk networks have good access to media and oxygen, assuring viability also for higher seeding densities than typically used for scaffold‐free spheroids, which are situated at the bottom of a well.

Together, our study shows that FN‐silk networks enable high‐throughput culture in commercially available plates, resulting in free‐floating and viable 3D engineered tissue constructs. This study thus supports the broad applicability and utility of the FN‐silk networks both when it comes to stem cell differentiation, and to maintaining cellular functions of specialized primary cells in a 3D set up, where it is important to keep a high viability throughout the culture, avoiding hypoxia and necrotic core formation.

## 4 Materials and Methods

Full details are available in the supplementary information, including Supplementary Table 1 and Supporting Information S1: Figures S1–S6.

### 4.1 Culture Formats

#### 4.1.1 FN‐Silk Network Cultures

FN‐silk networks were prepared (Figure 1) by mixing soluble FN‐silk (3 mg/mL in PBS, research batch with purity of 90%–95%, Spiber Technologies) with a newly prepared cell suspension. To avoid microspheres/silk aggregates, the FN‐silk solution was centrifuged at max speed (∼20,000 RCF) for 3 min before adding cells. A final FN‐silk concentration of 2 mg/mL and a cell concentration of either 2000, 3000 or 4000 cells/µL were used in the mix to get 10,000, 15,000 or 20,000 cells per foam respectively. For networks to be used in morphology studies by fluorescence microscopic imaging, 5% FN‐silk pre‐labeled with DyLight‐488 was added to the mixture. For networks in differentiation experiments, human recombinant laminin 421 (LN421, BioLamina) was added to a final concentration of 10 µg/mL in the silk‐cell mix. Culture medium was used for dilutions. One small drop of 5 µL FN‐silk‐cell mix was added to each well in one column of a U‐bottom ultra‐low attachment (ULA) 96‐well plate (Nunclon Sphera 174929) using a single channel pipette. Each drop was thereafter transformed into dense FN‐silk foams with integrated cells by rapid pipetting with a multichannel pipette (ca 30 strokes, around 2–3 strokes/s). This foaming procedure was repeated for each column to create the desired number of foams. The foams were thereafter stabilized for 10–20 min in a cell incubator. After the stabilization, 150–180 µL medium were added to the side of each well to detach the foams from the wells. The free‐floating foams were then either transferred with a spoon directly to new wells in a flat bottom 96‐well plate with medium or cultured overnight and transferred the following day. Over the following days in the cell incubator, the bubbles did gradually burst and the foams were transformed into FN‐silk networks. Medium changes for FN‐silk networks were performed every 2–3 days.

#### 4.1.2 Spheroid and 2D Control Cultures

Spheroids of MSCs were formed by seeding 96‐well ULA plates (Nunclon Sphera U‐bottom or Akura InSphero) with 3000 or 10,000 cells/well in 100 or 70 µL medium depending on plate type. PHH spheroids of 1500 cells were formed in 96‐well ULA plates (Corning) as described previously (Bell et al. 2016). Once PHH spheroids were sufficiently aggregated FBS was phased out and spheroids were cultured in serum‐free culture medium. Medium changes for all spheroids were performed every 2–3 days.

As 2D controls, hMSC and PHH were seeded as monolayers in TCT multiwell plates. For hMSCs, seeding densities of 10,000–15,000 cells/cm^2^ were used in various plate formats (6‐, 24‐ or 96‐well plates) coated with LN421 (10 µg/mL) or fibronectin (sigma F1141‐1MG, 50 µg/mL). PHHs were seeded and cultured in 2D by plating 80,000 cells/cm^2^ on a 48‐well plate.

### 4.2 Adipogenic Differentiation

hMSC (passage 3 or 4) were seeded to FN‐silk networks with or without addition of human recombinant laminin 421 (10 µg/mL, BioLamina), or as monolayers and spheroids, as described in section 4.1. FN‐silk networks were seeded with 20 000 cells, monolayers with 10,000–15,000 cells/cm^2^ in TCT plates coated with LN421 (10 µg/mL). Spheroids with 10,000 or 3000 cells in 70 µL/well were prepared in Akura plates (InSphero CS‐PB15) according to manufacturer's instructions.

Adipogenic differentiation was induced at around 80‐90% confluence for monolayers, and at Day 3 or 4 for 3D cultures, by changing from growth medium to Mesenchymal Stem Cell Adipogenic Differentiation Medium 2 (PromoCell C‐28016). Cells were cultured in differentiation medium with medium changes every second–third day for up to 3 weeks.

### 4.3 Data Analysis and Statistics

Results are presented as mean ± SEM unless otherwise specified. Statistical analyses were performed using Prism 9 (GraphPad, USA). For comparisons between two groups, unpaired two‐tailed heteroscedastic *t*‐tests were conducted with *n* ≥ 3 samples per group. For all other cases, 2‐way ANOVA was used. All data are included, with no outliers removed. Statistical significance was defined as *p* ≤ 0.05.

## Author Contributions

**Astrid Källén:** formal analysis, methods, investigation, visualization, writing – original draft. **Nayere Taebnia:** formal analysis, methods, investigation, visualization, writing – review and editing. **Mona Widhe:** conceptualization, methods, supervision, visualization, writing – review and editing. **Volker M. Lauschke:** funding acquisition, resources, supervision, writing – review and editing. **My Hedhammar:** conceptualization, funding acquisition, resources, supervision, writing – review and editing. All authors provided feedback on the work, and reviewed and proofread the final version of the article.

## Conflicts of Interest

V.M.L. is cofounder, CEO and shareholder of HepaPredict AB, as well as cofounder and shareholder of Shanghai Hepo Biotechnology Ltd. M.H. has shares in, and A.K. is employed by, Spiber Technologies AB, a company that aims to commercialize recombinant spider silk.

## Supporting information

Suppl Kallen rev clean.

## Data Availability

The data that support the findings of this study are available from the corresponding author upon reasonable request.
