# Supplementary material for: 3D Culture in Functionalized FN‐Silk Networks Facilitate Proliferation, Differentiation and Phenotypic Stability of Mature Human Primary Cells and Stem Cells
Source: Biotechnol Bioeng. 2025 Jun 17;122(9):2522–34. doi: 10.1002/bit.70002 (PMC12322619; doi:10.1002/bit.70002)
Supplement: Supplementary file 1 — Suppl Kallen rev clean. [file BIT-122-2522-s001.docx]

Supplementary information

# Supplementary materials and methods

## Cell culture

Human Bone Marrow-derived Mesenchymal Stem Cells (hMSC-bm #7500, ScienCell™) were maintained on human recombinant Laminin 521 (BioLamina, 5 µg/mL) coated tissue-culture treated (TCT) T75-flasks with Mesenchymal Stem Cell Growth Medium XF (PromoCell C-28019). GIBCO® Mouse (C57BL/6) Mesenchymal Stem Cells (mMSC) were maintained in TCT T75-flasks with DMEM/F-12 (Gibco 31330-038) supplemented with 5% FBS (Gibco 16140071) and 1% PenStrep. Both cell types were cultured in a cell incubator at 37°C and 5% CO_2_ and passaged at around 80-90% confluence using TrypLE™ Express Enzyme (Gibco™). Cell counting for experiments was done by either manual counting in Bürker chamber with trypan blue, automatic cell counting using TC20 cell counter (BioRad) or iPrasense Norma XS. Passages 3-9 were used for experiments with hMSC, and 22-36 for mMSC.

Cryopreserved primary human hepatocytes (PHH; Bioreclamation IVT, USA) were thawed following the supplier’s instructions and cultured as described under 4.1 Materials and methods in William’s E medium, supplemented with 11 mM glucose, 100 nM dexamethasone, 10 ng/mL insulin, 5.5 mg/L transferrin, 6.7 μg/L selenite, 2 mM L-glutamine, 100 U/mL penicillin, 0.1 mg/mL streptomycin, and 10% fetal bovine serum (FBS) during seeding.

## Cell seeding in FN-silk networks after foaming

An alternative method for seeding cells after foaming was also tested. This was done as described under 4.1.1 Materials and methods, but excluding cells in the FN-silk solution mix, and with the following additional steps after detaching the foams: the medium was removed and 5 µL cell suspension was seeded on top of each foam. After seeding, the foams were incubated for 25 min in the cell incubator to allow cell diffusion into the foam and attachment to FN-silk structures, before adding new culture medium.

## Cell viability and proliferation

Metabolic activity of 3D cultures was measured using CellTiter-Glo® 3D Cell Viability Assay (Promega G9683) in white 96-well plates according to the manufacturer’s instructions for mMSC. The protocol was slightly modified for hMSC measurements by an increased shaking time of 10 min at 900 rpm followed by a 15 min incubation at RT before recording luminescence in a plate reader (CLARIOstar, BMG Labtech). ATP standards were included in all measurements using rATP (Promega #E6011) diluted in culture medium to 10 µM, 5 µM, 1 µM and 0.1 µM. Samples were analyzed in triplicates and ATP standards in duplicates.

Live/Dead staining of 3D cultures was made using the LIVE/DEAD™ Viability/Cytotoxicity Kit (Invitrogen™ L3224). Cultures were incubated for 30 min with Calcein-AM (0.05%) and Ethidium-homodimer-1 (0.2%) in medium before imaging with fluorescence microscope Leica DM16000 B. Images were processed in ImageJ.

## Phalloidin staining of F-actin

Spheroids and FN-silk networks made with 5% 488-DyLight-labeled FN-silk added in the silk-cell mix were fixed with cold 4% paraformaldehyde (PFA) for 20 or 15 minutes respectively, followed by three PBS washes. Fixed samples were permeabilized with 0.2% Triton-X100 in PBS for 15 min and blocked with 5% donkey serum for 1 h before staining with Alexa Fluor 647 Phalloidin Molecular Probes (Cell Signaling Technology, #8940S, 1:70 or 1:100 dilution) for 30-60 min followed by nuclei counterstain with DAPI (Sigma, 1 µg/mL) for 10 min. Washes were done between each staining step with 0.2% Tween 20 in PBS. Stained samples were mounted in fluorescence mounting medium (DAKO) with iSpacers (SunjinLab #IS009 or #IS309) on microscope slides before imaging with fluorescence microscope Leica DM16000 or Stellaris 5 confocal microscope (Leica). Images were processed in ImageJ.

## Expression analysis

RNA extractions from hMSCs and differentiated MSCs cultured in networks, monolayers and spheroids were done using RNeasy micro or mini kits (Qiagen) with on-column DNase digestion. A single well was used for each RNA sample for monolayers (6 wp or 24 wp for mini and micro kit respectively). For networks seeded with 20 000 or 10 000 cells, 4 or 8 cultures were pooled to one RNA sample. Likewise, 18 or 24 spheroids, originally seeded with 3000 or 10000 cells respectively, were pooled to one RNA sample. Total RNA from PHHs cultured in networks, monolayers and spheroids was extracted using Qiazol lysis reagent (Qiagen).

The extracted RNA was then reverse transcribed into cDNA with SuperScript III reverse transcriptase (Invitrogen). RT-qPCR analysis was conducted using a TaqMan Universal Master Mix on a 7500 Fast Real-Time PCR system. Gene expression levels were calculated using the ΔΔCt method, with GAPDH serving as the housekeeping gene. A list of all primers used is available in Supplementary Table 1.

**Supplementary Table 1** - TaqMan primers used for gene expression analysis

| **Gene** | **Species** | **TaqMan Probe ID** |
| --- | --- | --- |
| GAPDH | Human | hs99999905_m1 |
| MKI67 | Human | hs01032443_m1 |
| CASP3 | Human | hs00234387_m1 |
| CA9 | Human | hs00154208_m1 |
| PPARG | Human | hs00234592_m1 |
| ADIPOQ | Human | hs00605917_m1 |
| FABP4 | Human | hs01086177_m1 |
| ALB | Human | hs00910225_m1 |
| CYP3A4 | Human | hs00604506_m1 |
| GLUL | Human | hs00365928_g1 |

## Differentiation read-outs

Metabolic activity measurements with CellTiter-glo 3D and Live/Dead staining of 3D cultures during the differentiation period was analyzed as described in supplementary section 1.3. RNA from differentiated samples and controls were extracted and analyzed with qPCR of the adipogenic markers Peroxisome proliferator-activated receptor gamma (PPARG), Adiponectin (ADIPOQ) and Fatty acid binding protein 4 (FABP4) as described under supplementary section 1.5.

### Lipid droplet staining

Lipid droplet staining of PFA fixed differentiation samples were done using either the fluorescent stain bodipy or the lysochrome dye Oil Red O. Bodipy staining was done by overnight incubation with 5 µM Bodipy-FL C12 (Invitrogen D3822), DAPI (sigma, 1 µg/mL) and 0.1% Tweeen-20 in PBS on a rocking table. The next day, samples were washed twice with PBS and networks were mounted with fluorescence mounting medium (dako) on microscope slides with iSpacers before imaging with an LSM880 confocal microscope (Zeiss). Oil Red O staining were done by firstly washing the samples with 60% isopropanol followed by staining for 15 min with freshly prepared working solution of Oil Red O. The working solution was prepared by diluting a 0.5% Oil Red O stock in isopropanol (CI 26125 or O1391 sigma) 3:5 with MilliQ, followed by filtering after standing 10 min at RT. Samples were then washed, firstly with 60% isopropanol followed by MilliQ, and cleared overnight in RapiClear 1.49 (SunJin Lab). Cleared samples were either imaged directly in the plate or mounted in RapiClear on microscope slides with iSpacers before imaging with a stereo microscope. Images were processed in ImageJ.

### AdipoRed Lipid quantification

Lipid quantification with AdipoRed (Lonza, #PT-7009) was done in triplicates on dissociated cells from FN-silk cultures and monolayers before and after differentiation. Cultures were washed with PBS - - and cells dissociated with 200 µL/well of 2.5% trypsin, no phenol red (gibco #15090046) for 30 min for 3D cultures and 5 min for monolayers in a cell incubator. Two networks were pooled in each sample well with trypsin and a single well was used for monolayers. After trypsinization, the cells were mixed vigorously to obtain a homogeneous suspension and 100 µL from each well were transferred to new assay wells in white plates with clear bottom (greiner 655098). The remaining volume of cell suspension was used for automatic cell counting in duplicates for each well using iPrasense Norma XS. 3.5 µL AdipoRed reagent was added to each assay well and mixed immediately, firstly with a pipette and then, after addition of reagent to all wells was completed, on a plate shaker (200 rpm, 5 min). Multiple fluorescence measurements with CLARIOstar plate reader were made starting from 10 min after reagent addition. Fluorescence intensity values were normalized against the number of cells in each sample.

### Adiponectin quantification with ELISA

Conditioned media were collected at the start and end of the differentiation, and frozen at −80 °C. To determine the adiponectin concentrations, an ELISA assay was performed according to the manufacturer’s recommended procedure (R&D Systems).

## Assessment of albumin secretion in PHH

Albumin levels in the culture media from PHHs were quantified using the Human Albumin ELISA Quantitation Set (Bethyl Laboratories, USA) according to the manufacturer's instructions. Supernatant samples were collected at each time point and immediately frozen at −80 °C until analysis. Albumin concentrations were determined by interpolation from the standard curve and normalized to the number of cells.

## Microscopy

Macroscopic images of 3D cultures were obtained using a Nikon stereo microscope (SMZ 745T). Fluorescence microscope Leica DM16000 B was used for imaging all live/dead stainings and the phalloidin/dapi stainings in supplement.

Confocal fluorescent images were acquired using an LSM880 confocal microscope (Zeiss, Germany) and Stellaris 5 confocal microscope (Leica, Germany). Images were post-processed with ImageJ Fiji analysis software.

## Size distribution

FN-silk networks and spheroids initially seeded with 10 000 hMSC were imaged over 1 week of culture using a top-view Nikon stereo microscope (SMZ 745T) with a 4X or 2X magnification. Area measurements of the cultures were made using ImageJ Fiji analysis software by freehand selections of the outer surface of the cultures.

# Supplementary figures

***Supplementary figure 1****. a) Representative* *images over 1 week culture of FN-silk networks (N=2, n=10-24) and spheroids (N=2, n=24) seeded with 10 000 hMSCs imaged with stereo microscope. Scale bars 300 µm. b) FN-silk networks (upper panel seeded with 10 000 mMSCs and imaged with stereo microscope, and spheroids seeded with 3000 (middle panel) or 10 000 (lower panel) mMSCs imaged with brightfield microscope. N=5, n=3-16. Scale bars 500 µm for networks and 100 µm for spheroids.) originally*

***
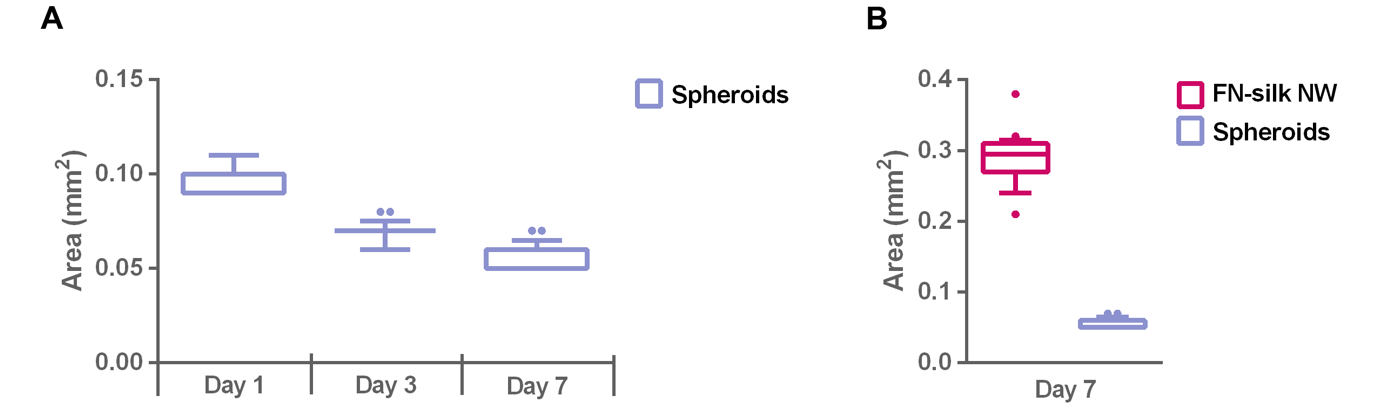
***

***Supplementary figure 2.*** *Size distribution plots of* ***a)*** *scaffold-free spheroids during one week of culture, and* ***b)*** *FN-silk networks and spheroids after 7 days culture, all initially seeded with 10 000 hMSCs/well. Area measurements were made in ImageJ from stereo microscope top view images (n=24).*


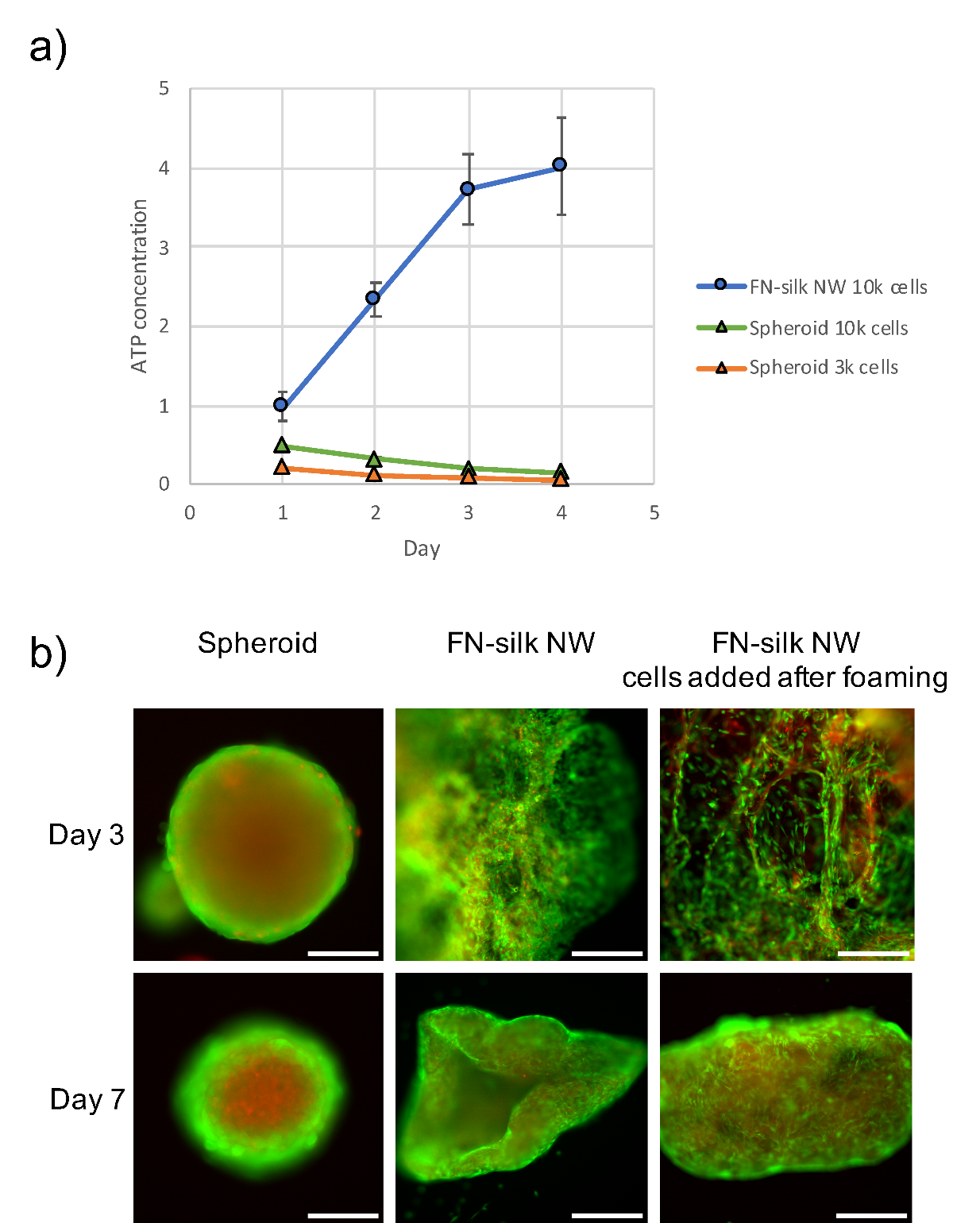


**Supplementary figure 3. Viability of mMSC in FN-silk networks and spheroids**. a) ATP levels as measured by CellTiter-Glo 3D Cell Viability assay of FN-silk networks initially seeded with 10,000 mMSCs (blue) and spheroids seeded with 10,000 (green) or 3000 mMSCs (orange), plot showing 1 of 3 independent experiments (n=3). **b)** Live (calcein, green) and dead (ethidium homodimer-1, red) staining of spheroids seeded with 10 000 mMSCs (left), and FN-silk networks seeded with 10 000 mMSCs before (middle) or after (right) foaming, imaged after 3 and 7 days of culture by fluorescence microscopy (N=5, n=2). Scale bars 300 µm for networks and 75 µm for spheroids.


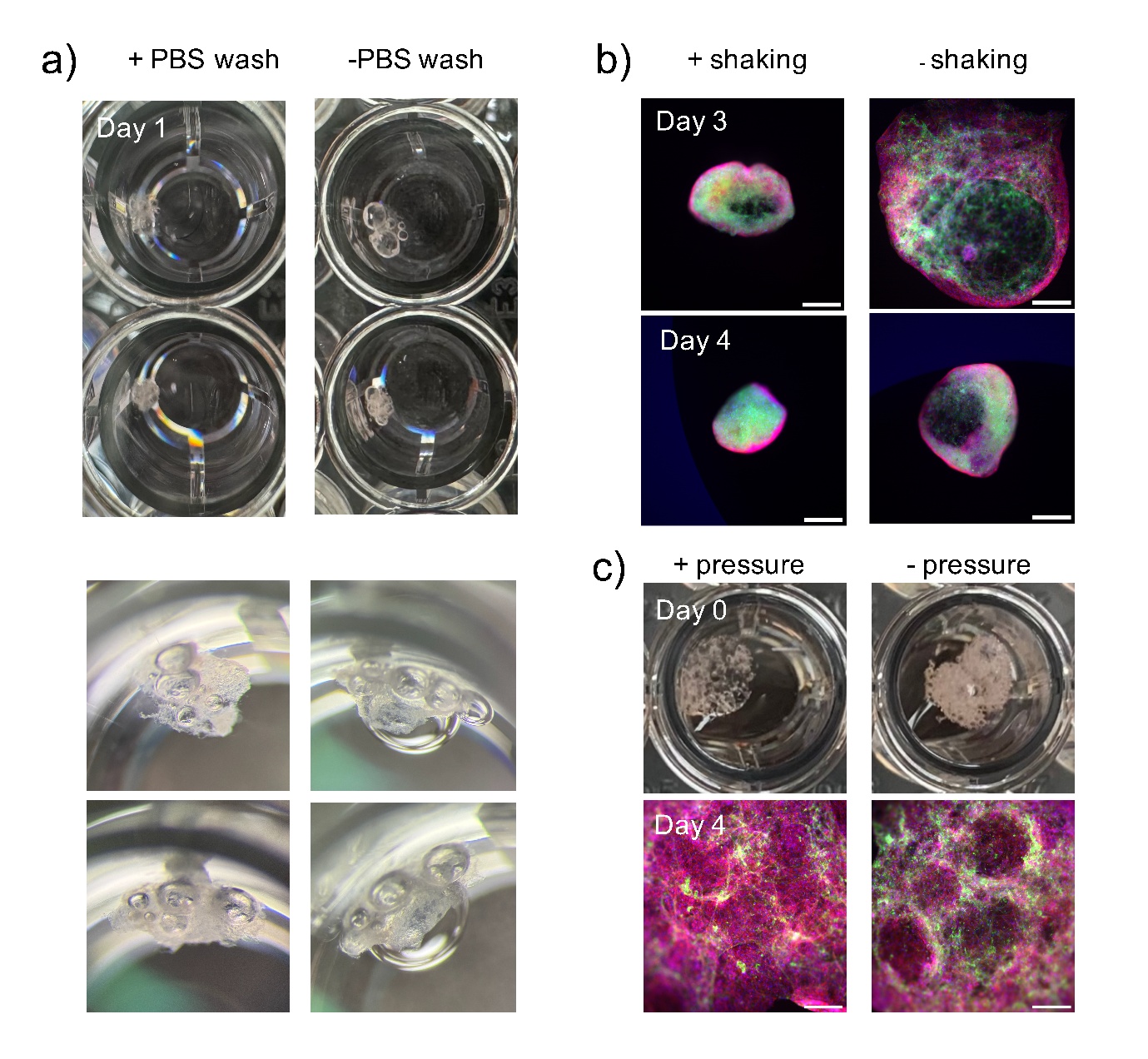


**Supplementary figure 4.** **Foam handling for expedited air bubble removal**. FN-silk networks seeded with 20 000 hMSC a) imaged at day 1 after and before PBS wash followed by medium change or, b) after 3 and 4 days of culture in incubator with or without shaking at 95 rpm before fixation and phalloidin (red) and DAPI (blue) staining with fluorescent FN-silk (green) included at seeding. c) FN-silk network seeded with 10 000 mMSC with and without pressure difference treatment at day 0 and after 4 days of culture, stained with phalloidin-Alexa Fluor 647 (red) and DAPI (blue) and DyLight-488 labeled FN-silk (green) included at seeding. Scale bars 400 µm.


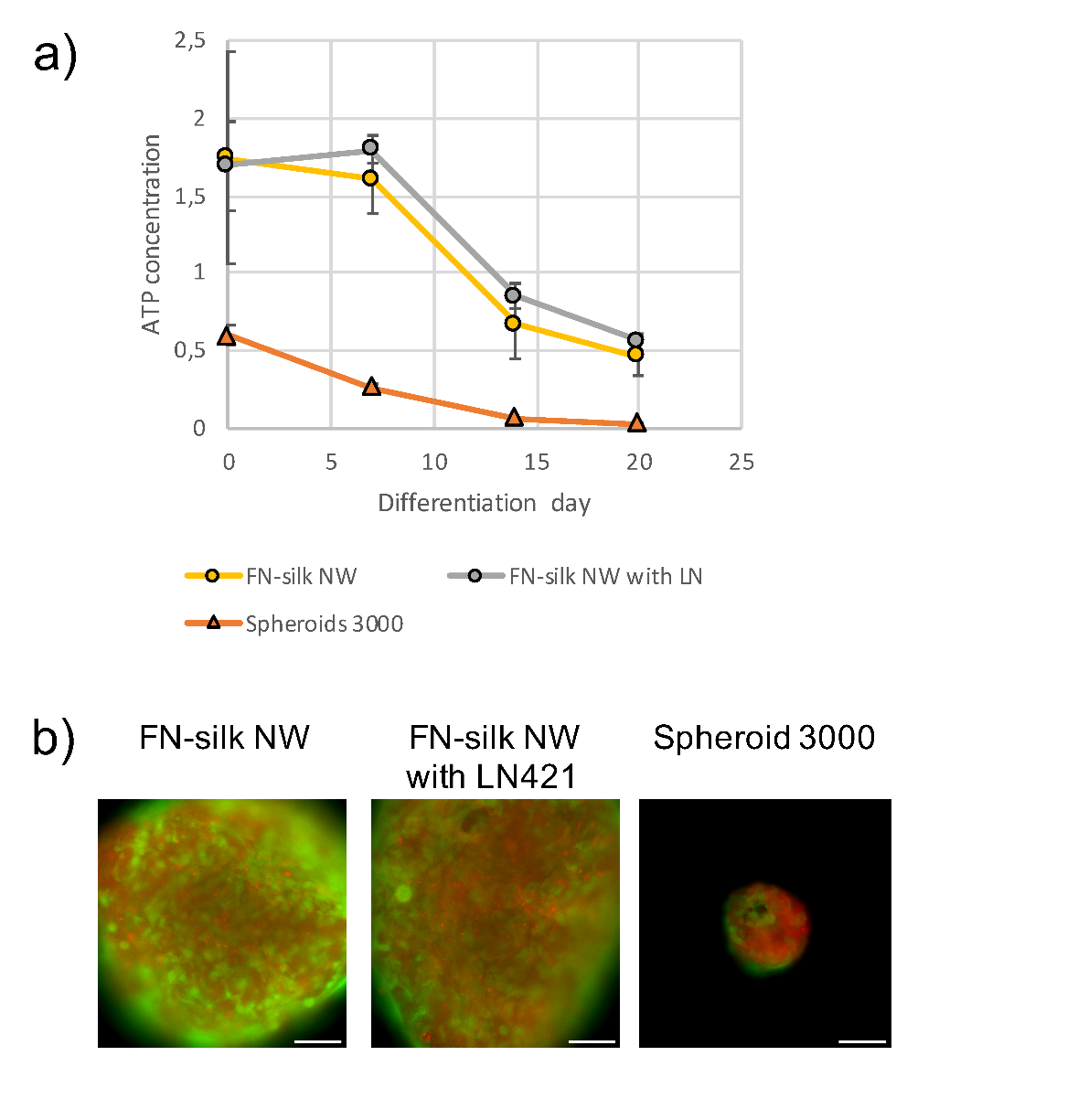


**Supplementary Figure 5. Viability during adipogenic differentiation of hMSC.** a) ATP levels, measured by CellTiter-Glo 3D Cell Viability assay (n=3), of hMSCs in FN-silk networks with (grey) or without the addition of LN421 (yellow), and spheroids (orange) during the 3-weeks long adipogenic differentiation. Day 0 is the differentiation start, i.e. culture day 3. b) Live (calcein, green) and dead (ethidium homodimer-1, red) staining of hMSCs in FN-silk networks (n=2) and spheroids (n=1) after 20 days of adipogenic differentiation, imaged by fluorescence microscopy. Scale bars 100 µm.


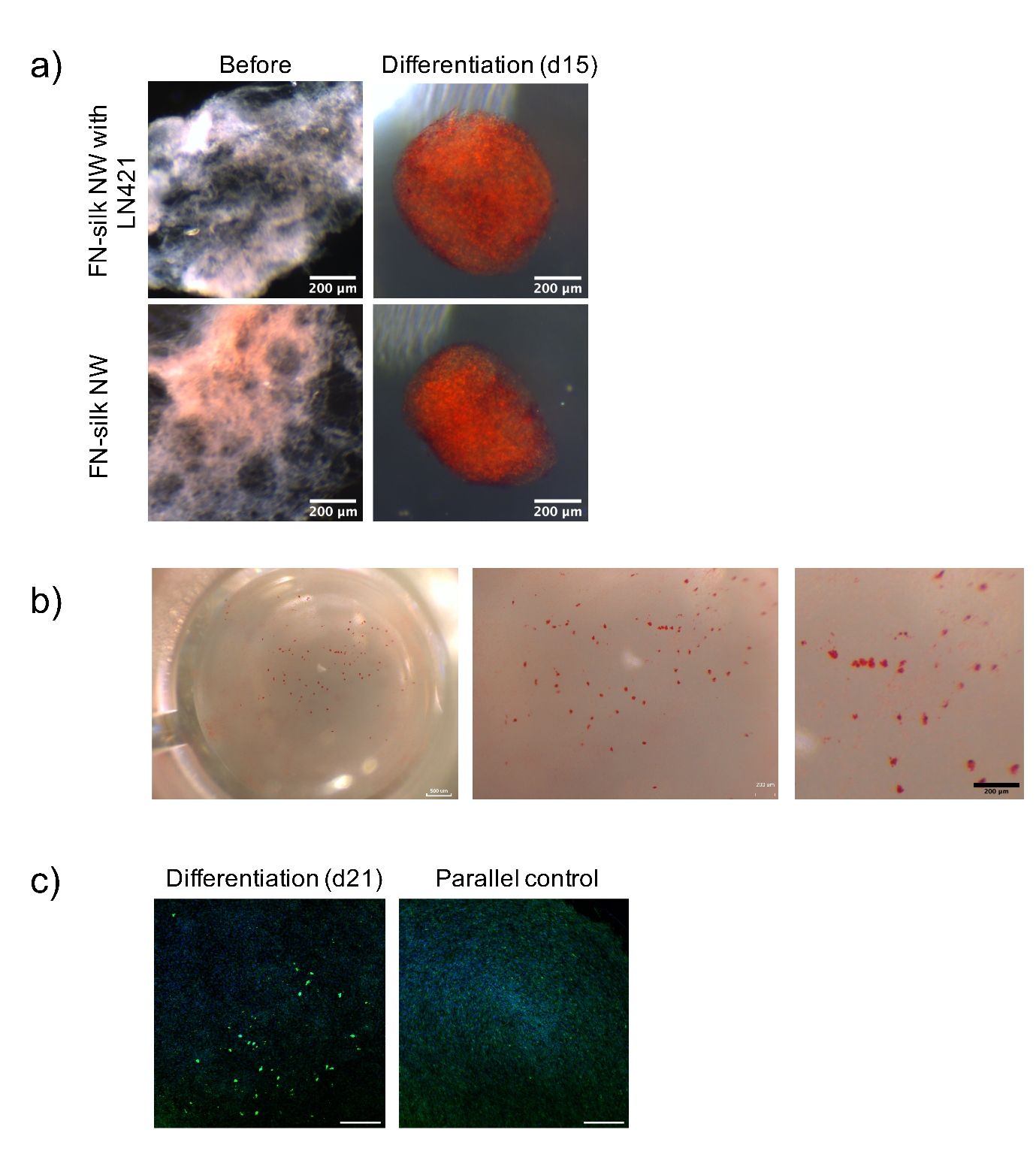


**Supplementary figure 6. Lipid droplet stainings.** a) hMSCs in FN-silk networks (NW) with or without the addition of LN421 fixed and stained with Oil Red O before and after 15 days of adipogenic differentiation. b), c) Lipid droplet stainings of hMSCs monolayer cultures after 3 weeks of adipogenic differentiation using b) Oil Red O as imaged by stereomicroscope or c) BODIPY® (4,4-difluoro-3a,4adiaza-s-indacene, green) and DAPI (counter stain, blue), imaged by confocal microscopy. Scale bar 200 µm in Oil Red O images and 500 µm in BODIPY images.
